# Optimized classification of potato leaf disease using EfficientNet-LITE and KE-SVM in diverse environments

**DOI:** 10.3389/fpls.2025.1499909

**Published:** 2025-05-02

**Authors:** Gopal Sangar, Velswamy Rajasekar

**Affiliations:** Department of Computer Science and Engineering, Faculty of Engineering and Technology, SRM Institute of Science and Technology, Vadapalani, Chennai, India

**Keywords:** EfficientNet-LITE, KE-SVM optimization, channel attention, 1-D local binary pattern, Sobel edge augmentation, uncontrolled environment data, potato leaf disease

## Abstract

**Introduction:**

Potatoes are a vital global product, and prompt identification of foliar diseases is imperative for sustaining healthy yields. Computer vision is essential in precision agriculture, facilitating automated disease diagnosis and decision-making through real-time data. Inconsistent data in uncontrolled contexts undermines classic image classification techniques, hindering precise illness detection.

**Methods:**

We present a novel model that integrates EfficientNet-LITE for enhanced feature extraction with KE-SVM Optimization for effective classification. KE-SVM Optimization cross-references misclassified instances with correct classifications across kernels, iteratively refining the confusion matrix to improve accuracy across all classes. EfficientNet-LITE improves the model's emphasis on pertinent features through Channel Attention (CA) and 1-D Local Binary Pattern (LBP), while preserving computational economy with a reduced model size of 12.46 MB, fewer parameters at 3.11M, and a diminished FLOP count of 359.69 MFLOPs.

**Results:**

Before optimization, the SVM classifier attained an accuracy of 79.38% on uncontrolled data and 99.07% on laboratory-controlled data. Following the implementation of KE-SVM Optimization, accuracy increased to 87.82% for uncontrolled data and 99.54% for laboratory-controlled data.

**Discussion:**

The model's efficiency and improved accuracy render it especially appropriate for settings with constrained computational resources, such as mobile or edge devices, offering substantial practical advantages for precision agriculture.

## 1 Introduction

Crop and plant diseases lead to substantial revenue drops, incurring elevated disease management expenses and financial losses for farmers globally. Potatoes serve as a fundamental food source in India, which ranks as the second-largest producer globally, contributing over 15% to worldwide potato production. In India, potatoes are grown on around 2 million hectares, yielding 56 million tons (Mishra et al., 2024), thereby playing a crucial role in food security and the economy of agriculture. Potato crops experience yield losses of 5% to 15% owing to leaf diseases (Mishra et al., 2024), necessitating the implementation of effective disease management methods. Precisely diagnosing and categorizing diseases under diverse conditions is important for effective disease management. Conventional methods (Singla et al., 2024) necessitated manual field scouting, resulting in delayed disease diagnosis. These approaches are both inefficient and subjective, depending on visual evaluations conducted by trained plant pathologists. Computer vision-based image analysis (Gulame et al., 2023; Tholkapiyan et al., 2023) has been developed to address these constraints, enabling rapid and precise disease identification. However, initial solutions primarily focused on feature engineering to define particular attributes for each illness, which is unfeasible for the extensive variety of plant species and diseases. This has concluded in increased dependency on deep learning (DL) to provide more generalized and scalable options.

In recent years, deep learning has gained prominence because to developments in Graphics Processing Units (GPUs), increased storage space, and the availability of vast datasets. Convolutional Neural Networks (CNNs) (Huang et al., 2023) have become highly favored for the recognition and classification of plant diseases owing to their capacity to independently extract and learn optimal features from images. Although they perform well in controlled settings, numerous models fail to reproduce these outcomes with field data acquired under uncontrolled conditions (Shabrina et al., 2024). To mitigate this deficiency, the EfficientNet-LITE model, based on Convolutional Neural Networks (CNN) (Haque et al., 2022; Khamparia et al., 2020; Nagaraju and Chawla, 2022; Thakur et al., 2022), was utilized to extract pertinent and advanced features from images, facilitated by the incorporation of Channel Attention (CA) (Chen et al., 2021) and 1-D Local Binary Pattern (LBP) (Rachmad et al., 2022) features. The incorporation of 1-D LBP for texture analysis from feature maps is a distinctive method that markedly improved the model’s capacity to identify complex patterns in uncontrolled settings. Additionally, Sobel edge-detected samples were incorporated into the improved dataset, providing an innovative method to improve edge information during training. Furthermore, KE-SVM Optimization (Deepti, 2023; Shrivastava et al., 2023) was employed to enhance classification by optimizing (Sorensen and Nielsen, 2018) SVM kernels and producing superior prediction data. This integrated methodology attained elevated precision in both regulated laboratory settings and demanding outdoor environments. The primary contributions of the paper are outlined below.

- The EfficientNet-LITE model, with the innovative incorporation of Channel Attention and the original utilization of 1-D Local Binary Pattern features, substantially enhanced the accuracy of plant disease classification, especially in severe uncontrolled situations. This distinctive integration enabled the model to concentrate more efficiently on pertinent image attributes.
- The incorporation of Sobel edge-detected samples into the supplemented dataset greatly enhanced the model’s capacity to capture and leverage edge information, consequently raising classification performance.
- The KE-SVM Optimization utilized a kernel ensemble and presented an innovative method to enhance the confusion matrix by revisiting misclassified samples and accurately categorizing them with other kernels. This novel approach successfully reduced the constraints of conventional SVMs, resulting in enhanced classification efficiency across various datasets.
- The integration of EfficientNet-LITE with KE-SVM Optimization demonstrated a revolutionary methodology that attained higher accuracy and resilience. The model effectively generalized over both controlled and uncontrolled datasets.
- This research introduced an innovative, rapid, precise, and dependable approach for classifying plant diseases, thereby enhancing agricultural disease management, potentially reducing yield losses, and enabling informed decision-making for farmers.

Effective management of plant diseases requires timely and precise identification and classification. Development in artificial intelligence and machine learning has resulted in substantial enhancements in automated disease detection. This review examines contemporary methodologies and technologies, concentrating on image processing and deep learning models applied to various crops, with the objective of summarizing current achievements and pinpointing research opportunities.

Nabila Husna Shabrina et al. revealed shortcomings in the PlantVillage dataset for the diagnosis of potato leaf diseases in real-world scenarios. To resolve this, they presented a novel dataset of 3,076 pictures obtained in uncontrolled settings, encompassing seven disease varieties. This dataset offers a more precise depiction of potato leaf conditions. Testing EfficientNetV2B3 (Shabrina et al., 2024) resulted in 73.63% accuracy on the new dataset, in contrast to 98.15% on PlantVillage.

Aanis Ahmad et al. investigated (Ahmad et al., 2023) the generalization capacity of deep learning (DL) models for diagnosing corn diseases in field conditions using many datasets, including PlantVillage, PlantDoc, Digipathos, NLB, and a proprietary CD&S dataset. Five deep learning architectures—InceptionV3, ResNet50, VGG16, DenseNet169, and Xception—were trained utilizing diverse dataset pairings. DenseNet169 exhibited enhanced performance, achieving an accuracy of 81.60% using RGBA images from the CD&S dataset after background removal. Furthermore, the amalgamation of field-acquired and laboratory data, encompassing sources from PlantVillage and CD&S, yielded an accuracy range of 77.50% to 80.33%, hence improving model generalization for field application.

Penghui Gui et al. tackled the issue of identifying plant diseases in uncontrolled field environments. They proposed an enhanced CNN model for field plant (Gui et al., 2021) disease identification (FPDR), incorporating strategies such as backdrop substitution and leaf resizing to optimize data augmentation. To improve feature differentiation, they employed a channel orthogonal constraint and utilized species categorization as a supplementary task. Utilizing the proprietary Field-PlantVillage (Field-PV) dataset, comprising 665 field photos, the model attained an accuracy of 72.03%, representing a substantial enhancement from 41.81%, despite being exclusively trained on the PlantVillage dataset.

A. Ubaidillah et al. sought to improve the categorizing of corn diseases using Random Forest, Neural Network, and Naive Bayes (Ubaidillah et al., 2022) techniques. The study utilized a compilation of corn leaf photographs obtained from agricultural regions in the Madura Region, concentrating on four classifications: healthy, gray leaf spot, blight, and common rust. The Neural Network technique outperformed the alternatives, with an AUC of 90.09%, a classification accuracy of 74.44%, an F1-score of 72.01%, precision of 74.14%, and recall of 74.43%, so establishing it as the most effective model for detecting maize diseases.

Priyanka Sahu and associates proposed a Deep-Dream (DD) architecture (Sahu et al., 2023) for Crop Leaf Disease Detection (CLDD), amalgamating deep learning (DL) with machine learning (ML) techniques. The study utilized the tomato crop dataset from PlantVillage and created 24 Hybrid Deep Neural (HDN) models, utilizing EfficientNet (B0-B7) as a feature extractor in conjunction with classifiers such as Random Forest (RF), AdaBoost (ADB), and Stochastic Gradient Boosting (SGB). The DD-EffiNet-B4-ADB model achieved optimal accuracy, ranging from 84% to 96%.

Hieu Phan et al. presented a deep learning approach utilizing Simple Linear Iterative Clustering (SLIC) segmentation (Phan et al., 2022) to identify diseased regions on corn leaves. The study employed five pre-trained models—VGG16, ResNet50, DenseNet121, Xception, and InceptionV3—on the PlantVillage and CD&S datasets, concentrating on super-pixel classes like northern leaf blight, gray leaf spot, and common rust. One hundred models were trained using diverse segments and split ratios. DenseNet121 achieved a peak accuracy of 97.77% on the CD&S dataset, employing five segments per image and an 80:20 split. Web and mobile applications were developed for disease identification, demonstrating the effectiveness of automated disease tracking relative to manual monitoring.

Mohit Agarwal et al. devised an efficient CNN model of 8 hidden layers (Agarwal et al., 2020) for the identification of tomato illnesses, therefore alleviating the computational demands linked to pre-trained models. Their approach, assessed with the PlantVillage dataset, achieved an accuracy of 98.4%, surpassing traditional machine learning methods (94.9% with k-NN) and pre-trained models like VGG16 (93.5%). The research employed image pre-processing techniques to enhance efficiency, achieving an accuracy of 98.7% on additional datasets. This study highlights the effectiveness and efficiency of lightweight (Zhu et al., 2023) CNN (Dai et al., 2023) models for disease detection in tomato crops.

Hasibul Islam Peyal and associates developed a lightweight 2D CNN model employing deep learning for the categorization of diseases in tomato and cotton plants. The algorithm, incorporated into an Android application named “Plant Disease Classifier,” (Peyal et al., 2023) proficiently categorized 14 classifications, consisting of 12 diseased and 2 healthy categories. Despite having fewer variables than pre-trained models like VGG16, VGG19, and InceptionV3, it achieved an impressive average accuracy of 97.36%, with precision, recall, and F1-scores around at 97%, and an Area under Curve (AUC) score of 99.9%. The utilization of Grad-CAM for visual interpretations and the model’s rapid classification time of around 4.84ms highlight its efficiency and effectiveness in disease detection.

Qiang Dai et al. created DATFGAN, a generative adversarial network that employs dual-attention and topology-fusion techniques to enhance the identification of agricultural disease photos. DATFGAN (Dai et al., 2020) improves image clarity and resolution, alleviating issues related to unclear images that hinder identification accuracy. The network’s weight-sharing approach reduces the parameter count, and actual evidence demonstrates that DATFGAN produces visually superior results and significantly outperforms existing methods in practical identification tasks.

Junde Chen et al. developed the Crop Disease Recognition Model (CDRM), including the Location-wise Soft Attention mechanism (Ubaidillah et al., 2022) into a pre-trained MobileNet-V2 to enhance the detection of subtle lesion features. This model addresses challenges associated with chaotic backgrounds and variable lighting in crop disease images. The study’s experimental results demonstrated an average accuracy of 99.71% on an open-source dataset, with a 99.13% accuracy in challenging conditions. The proposed method outperforms prior dominant techniques, showcasing its effectiveness and robustness in detecting agricultural illnesses.

Rabbia Mahum et al. proposed an enhanced deep learning technique for the diagnosis and categorization of potato leaf diseases. Unlike existing methods that categorize potato leaves into two groups utilizing the Plant Village dataset, their approach classifies leaves into five separate categories: Potato Late Blight (PLB), Potato Early Blight (Feng et al., 2023) (PEB), Potato Leaf Roll (PLR), Potato Verticillium Wilt (PVw), and Healthy (PH). Their model achieved an accuracy of 97.2% by utilizing a pre-trained Efficient DenseNet (Mahum et al., 2022) model, integrating an additional transition layer, and implementing a reweighted cross-entropy loss function. This method effectively tackles class imbalance and overfitting, offering a robust solution for comprehensive disease classification in potato leaves.

Zubair Saeed and associates developed a deep learning system focused on computer vision for the early detection and classification of potato leaf diseases. Utilizing deep convolutional neural networks (Saeed et al., 2021), specifically ResNet-152 and InceptionV3, trained on the Kaggle potato dataset, their methodology achieved accuracies of 98.34% and 95.24%, respectively, with a learning rate of 0.0005. The method precisely classifies potato leaves into three categories: healthy, early blight, and late blight. This method aims to mitigate economic losses by enabling the prompt detection of disease outbreaks through accurate image-based categorization.

Kashif Shaheed et al. developed EfficientRMT-Net, a novel model that combines Vision Transformer (ViT) with ResNet-50 (Shaheed et al., 2023) for the automated detection and classification of potato leaf diseases. This technology addresses the limitations of traditional methods, such as labor-intensive procedures and inadequate illness detection. EfficientRMT-Net utilizes CNN for feature extraction, depth-wise convolution to reduce processing demands, and a stage block architecture to enhance scalability and sensitivity. The model, trained on bespoke datasets, achieved accuracies of 97.65% on a generic dataset and 99.12% on a tailored potato leaf dataset. EfficientRMT-Net offers a dependable approach for accurate disease classification, consequently improving crop yield and resource efficiency.

Mingjie Lv and associates devised a maize leaf disease recognition method to tackle challenges including variable lighting and complexities in feature extraction. Their methodology integrates a maize leaf enhancement framework and the DMS-Robust AlexNet, an advanced neural network (Lv et al., 2020) based on AlexNet. This network incorporates dilated and multi-scale convolutions to improve feature extraction. It utilizes batch normalization to reduce overfitting, with the PReLU activation function and Adabound optimizer to improve convergence and precision. Experimental results demonstrate that this technique significantly enhances disease identification in complex scenarios, providing a dependable alternative for advanced plant disease diagnostics.

Hatice Catal Reis and Veysel Turk developed the Multi-head Attention Mechanism Depthwise Separable Convolution Inception Reduction Network (MDSCIRNet) for the early identification of potato leaf diseases. This deep convolutional neural network utilizes depthwise separable convolutions and a multi-head attention mechanism to enhance classification accuracy. MDSCIRNet (Reis and Turk, 2024) achieved an accuracy of 99.33% by combining deep learning with SVM, outperforming contemporary algorithms such as Xception and MobileNet, as well as traditional methods like SVM and Random Forest. The study highlights the effectiveness of MDSCIRNet in improving early disease detection and reducing financial losses for agricultural producers.

Xiangpeng Fan and Zhibin Guan address critical challenges in maize disease identification with their proposed VGNet, a system that employs a pretrained VGG16 model. VGNet incorporates batch normalizing, global average pooling, and L2 normalization to enhance performance. Utilizing transfer learning and the Adam optimizer, the model achieves an accuracy of 98.3% with a learning rate of 0.001, exhibiting remarkable precision and recall for nine maize diseases. VGNet’s small architecture (Fan and Guan, 2023), requiring only 79.5 MB, enables efficient processing, demonstrating effective disease recognition with a testing duration of 75.21 seconds for 230 images.

The reviewed literature demonstrates significant advancements in plant disease classification learning models, (Saritha and Thangaraja, 2023; Shahoveisi et al., 2023) using deep learning and machine learning models, yet several limitations persist. Many studies rely heavily on the PlantVillage dataset, which, while comprehensive, is collected in controlled environments and lacks diversity for real-world applications. For instance, Nabila Husna Shabrina et al. and Penghui Gui et al. highlighted the challenges of generalization in uncontrolled settings. Additionally, while methods such as DenseNet and EfficientNet have been explored, the absence of innovative feature extraction techniques, such as attention mechanisms and edge detection, limits their performance in detecting fine-grained features. Furthermore, traditional classifiers like SVMs, as used by A. Ubaidillah et al., often suffer from limitations in handling misclassified samples, reducing overall efficiency. Despite efforts to enhance accuracy, many studies fail to effectively combine lightweight models with robust optimization techniques for scalable and practical applications.

The proposed methods address these gaps by introducing EfficientNet-LITE with Channel Attention (Haider et al., 2024; Kumar et al., 2023; Navrozidis et al., 2018) and 1-D Local Binary Pattern (LBP) features, enabling precise focus on critical attributes even in uncontrolled environments. The inclusion of Sobel edge-detected samples enhances fine-detail recognition, while KE-SVM Optimization revisits and corrects misclassified samples, significantly improving classification efficiency. This integrated approach achieves superior generalization across diverse datasets, offering a fast, accurate, and reliable solution for real-world agricultural disease management, ultimately empowering farmers to reduce yield losses.

The remainder of the article is organized as follows: Section 2 outlines the structure of the feature extraction and classification model. Section 3 examines the experimental findings and analysis, while Section 4 presents the conclusions and future directions.

## 2 Materials and methods

**Figure 1:**
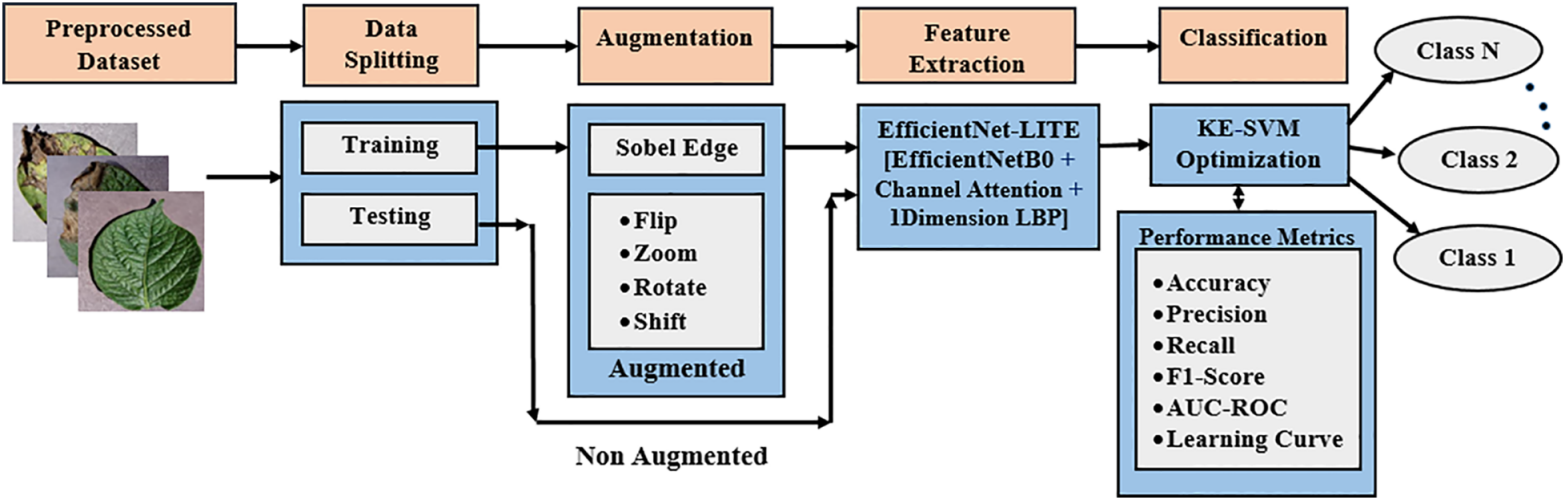
Proposed methodology for potato leaf disease classification.

The proposed approach initiates with image augmentation and Sobel edge identification to improve and diversity the dataset. **Figure 1** illustrates the application of an attention-based EfficientNet-LITE model for feature extraction to identify essential leaf attributes, succeeded by KE-SVM optimization for precise classification of potato leaf diseases across diverse environments.

### 2.1 Dataset collection

**Figure 2:**
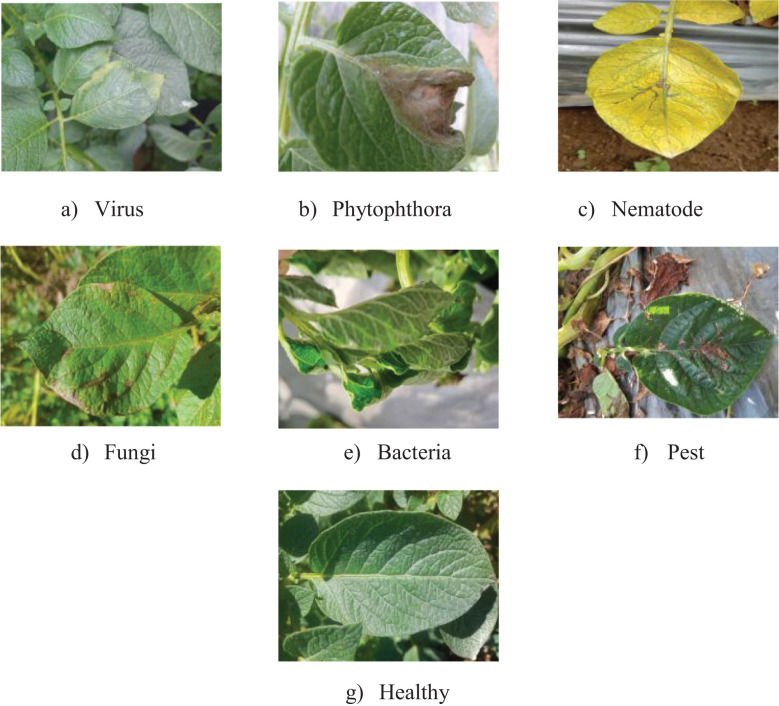
Samples of the seven categories in the potato leaf disease dataset: **(a)** Virus, **(b)** Phytophthora, **(c)** Nematode, **(d)** Fungal, **(e)** Bacteria, **(f)** Pest, and **(g)** Healthy.

This work utilized two datasets for the detection of potato leaf diseases: one from an uncontrolled environment (Shabrina et al., 2024) in Indonesia and the PlantVillage Dataset (Potato Species) (Shaheed et al., 2023) from a controlled laboratory setting. The first dataset, acquired from a Kaggle source, was compiled from multiple potato farms throughout Java Island by teams from Universitas Multimedia Nusantara and Universitas Gadjah Mada. It comprises 3,076 photos categorized into seven disease types: **Figure 2** (a). virus, (b). phytophthora, (c). nematode, (d). fungal, (e). bacteria, (f). pest, and (g). healthy, taken under various settings. **Figure 2** presents the sample photographs for each class. Each image possesses a resolution of 1500 × 1500 pixels and is stored in.jpg format for accessibility and compatibility with image-processing software.

The second dataset, PlantVillage (potato species), has 2,152 photos categorized into three classes: Healthy, Potato Late Blight, and Potato Early Blight, captured under uniform lighting circumstances with a resolution of 256 x 256 pixels. Both datasets provide a significant contrast between real-world and controlled settings for assessing model efficacy in disease diagnosis.

### 2.2 Preprocessing

Use bilinear interpolation (cv2.INTER_LINEAR) (Shabrina et al., 2024) to resize 1500x1500 potato leaf disease images to 224x224 pixels for machine learning models. This scaling was necessary to match image dimensions to models. We picked bilinear interpolation because it smoothed images while maintaining crucial characteristics and particulars from the high-resolution originals. Preprocessing the potato leaf disease images reduced computational effort and memory utilization, optimizing model performance and preparing the dataset for training and evaluation.

### 2.3 Data augmentation strategy

A complete data augmentation technique was applied to expand the training dataset of potato leaf disease image and improve the performance and resilience of the machine learning model. The initial dataset consisted of 3,076 pictures, with 2,460 allocated for training and 616 left aside for testing. Various augmentation strategies were employed to generate a more diverse and comprehensive training dataset, substantially enhancing the quantity of training samples.

Multiple fundamental augmentation methods were employed (Shabrina et al., 2024) to synthetically enlarge the training dataset. Rotation within a 20-degree range was implemented to imitate diverse viewing angles, enhancing the model’s capacity to generalize across multiple orientations. Width and height adjustments of up to 20% of the image dimensions were executed to simulate differences in image positioning. Furthermore, shear transformations with a magnitude of 0.2 were implemented to produce tilting effects, facilitating the model’s ability to manage images with perspective deviations. Zoom changes, with modifications of up to 20%, emulated various focal lengths and scales. Horizontal flips were utilized to mirror pictures and augment the model’s resilience to variations in orientation.

Sobel edge detection was employed to enhance the edges and transitions in the potato leaf disease images. Employing the OpenCV library, Sobel filters calculated gradients in both the x and y directions, yielding edge-detected representations of the source images. This technique enhanced texture and boundary information, which was integrated into the training dataset. The edge-detected images were merged with the augmented versions generated through fundamental changes, enhancing the dataset with intricate edge information.

**Figure 3:**
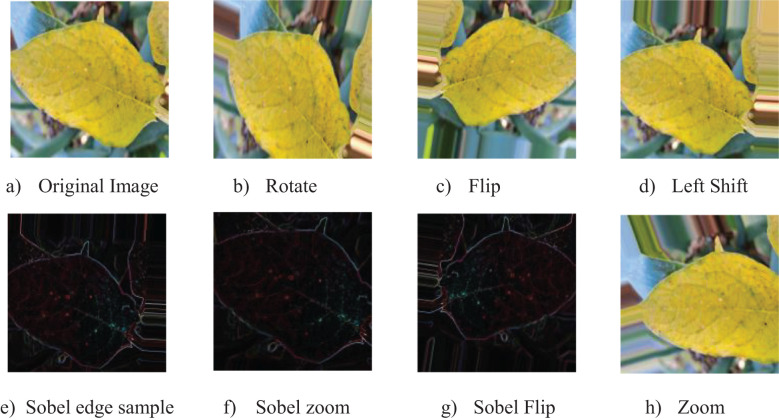
Sample images demonstrating original and augmented versions using various techniques: **(a)** Original Image, **(b)** Rotate, **(c)** Flip, **(d)** Left Shift, **(e)** Sobel Edge Sample, **(f)** Sobel Zoom, **(g)** Sobel Flip, and **(h)** Zoom.

The enhancing method was efficiently performed by processing images of potato leaf disease in phases. Each image in a batch was initially converted to float32 format and augmented to incorporate a batch dimension. Six specific augmentations were done to each image with Keras’s ImageDataGenerator class, enabling transformations including rotation, shifting, shearing, zooming, and flipping. Furthermore, Sobel edge detection was executed to produce further variations. **Figures 3a–h** illustrates the modified photos, accompanied by their respective labels, image names, and class names, which were subsequently gathered and preserved for model training.

This augmentation method led to a significant increase in the quantity of training samples. The initial training dataset of 2,460 photos was enlarged to 14,760 augmented samples (Xiong et al., 2020), incorporating those enhanced by Sobel edge detection. The quantity of original testing samples stayed at 616 and was not increased. The augmentation of the training dataset yielded a more varied collection of images, markedly improving the model’s capacity to generalize and excel in multiple circumstances.

### 2.4 Feature extraction

EfficientNet-LITE is an enhanced version of the basic EfficientNetB0 (Upadhyay et al., 2024) design, specifically engineered to improve feature extraction through the strategic integration of a Channel Attention (CA) mechanism and 1-D Local Binary Pattern (LBP) for features. The improvements implemented post-Global Average Pooling layer are designed to augment the model’s capacity to concentrate on pertinent features in images of diseased potato leaves, thus improving performance while preserving computational efficiency.

**Figure 4:**
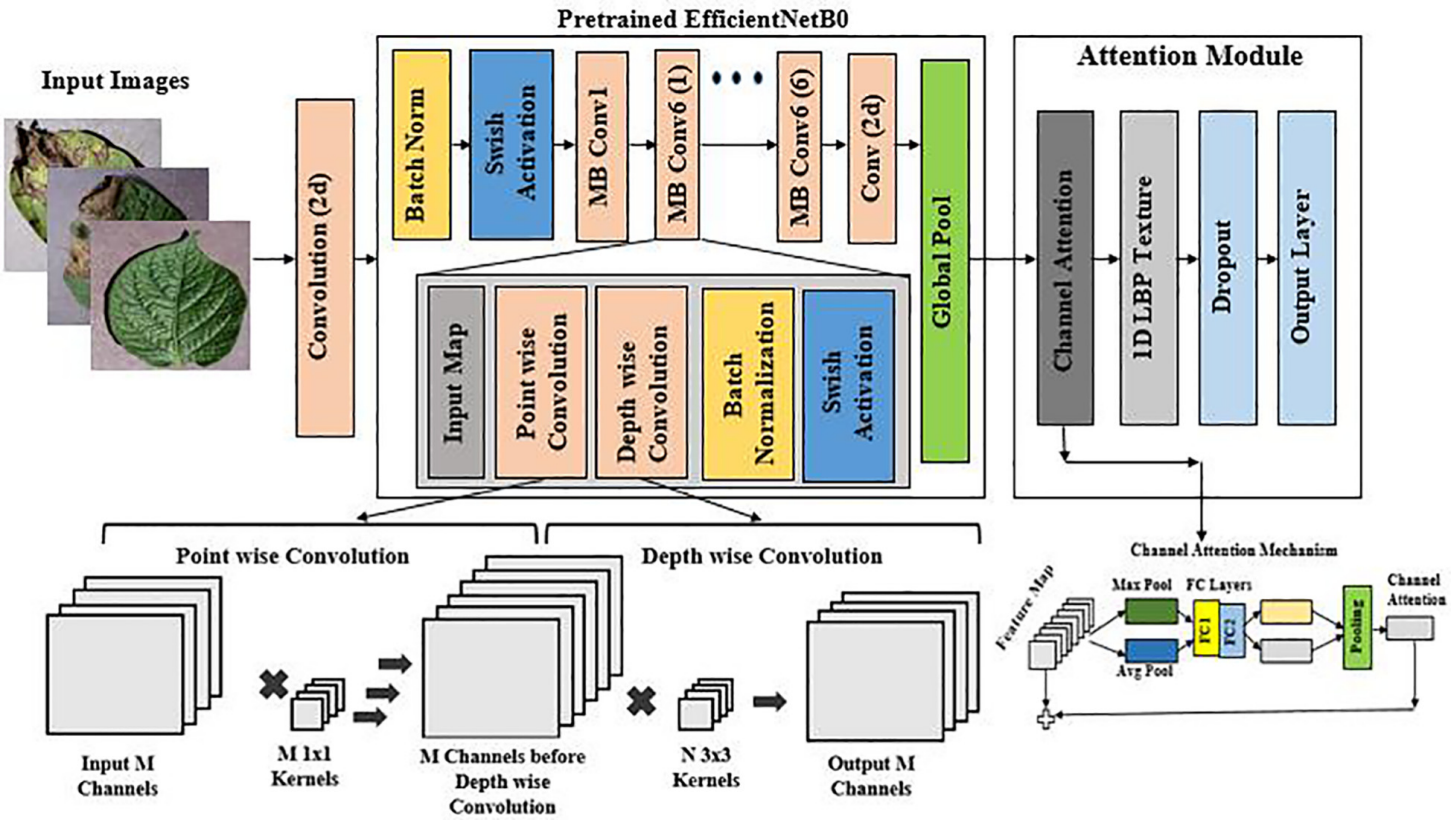
The architecture of EfficientNet-LITE Model with channel attention mechanism.

EfficientNet-LITE preserves the key principles of EfficientNetB0, which optimizes network depth, width, and resolution for enhanced accuracy with reduced parameters and FLOPs, while incorporating an attention mechanism for more targeted feature extraction. **Figure 4** (Reproduced from (Upadhyay et al., 2024)) shows the combination of EfficientNetB0 with Channel Attention mechanism. In contrast to EfficientNetB0, which depends exclusively on convolutional processes and depthwise separable convolutions (Reis and Turk, 2024), EfficientNet-LITE’s incorporation of Channel Attention and 1-D LBP enables the network to dynamically emphasize significant features. This produces a model that is both efficient and proficient at identifying nuanced patterns and details in potato leaf images, rendering it especially suitable for jobs demanding high accuracy with constrained computational resources.

The incorporation of the Channel Attention mechanism with 1-Dimensional LBP in EfficientNet-LITE tackles certain issues in feature extraction.

#### 2.4.1 Channel Attention (CA)

Channel Attention operates by initially condensing the spatial dimensions of the input tensor into a channel descriptor by global average pooling. This description encapsulates the overall context for each channel, succinctly conveying its significance.


(1) $$z_{c}=\frac{1}{H*W}\sum_{h=1}^{H}\sum_{w=1}^{W}X_{b,c,h,w\;}$$

In Equation 1, $z_{c}$ the global average is pooled value for channel *c*, $X_{\text{b},\text{c},\text{h},\text{w}}$ is the value of the input tensor at batch b, channel c, height h, and width w.

The two completely connected layers subsequently convert this description into a series of attention weights. The initial fully connected layer diminishes the descriptor’s dimensionality, whereas the subsequent fully connected layer reverts it to the original channel dimension. The ReLU activation introduces non-linearity, while the sigmoid activation guarantees that attention weights remain constrained between 0 and 1.

The vector *z* is then passed through two fully connected (FC) layers to generate channel attention weights:


(2) $$\text{First FC Layer}:\;y_{1}=ReLU(W_{1}z+b_{1})$$

In Equation 2, $W_{1}$ is the weight matrix of the first fully connected layer, $b_{1}$ is the bias vector of the first fully connected layer, ReLU is the Rectified Linear Unit activation function.


(3) $$\text{Second FC Layer}:\;y_{2}=W_{2}y_{1}+b_{2}$$

In Equation 3, $W_{2}$ is the weight matrix of the second fully connected layer, $b_{2}$ is the bias vector of the second fully connected layer.

Apply a sigmoid activation function to obtain the channel attention weights:


(4) $$a_{c}=\sigma (y_{2})$$

In Equation 4, σ is the sigmoid function, $a_{2}$ is the attention weight of channel c.

Ultimately, these attention weights are employed to scale the original input tensor, accentuating channels with greater weights and reducing the influence of channels with lesser weights. This approach allows the model to concentrate on the most pertinent aspects, enhancing its capacity to derive significant information from the incoming data.

#### 2.4.2 1-D Local Binary Pattern (1D LBP):

1-D Local Binary Pattern (1-D LBP) is a method for identifying textural features from one-dimensional data, such sequential signals or feature vectors obtained from photographs. It operates by juxtaposing each data point with its adjacent counterparts to produce a binary pattern, subsequently transformed into a decimal code. The codes are compiled into a histogram that illustrates the distribution of local textures within the data points. This approach is resilient to periodic changes and effectively identifies critical local structures, including edges and peaks. The 1-D LBP (**Algorithm 1**) histogram offers a concise and distinctive feature descriptor that is efficient for signal classification and texture analysis tasks.

Algorithm 1 **1–D Local Binary Pattern (1–D LBP).**

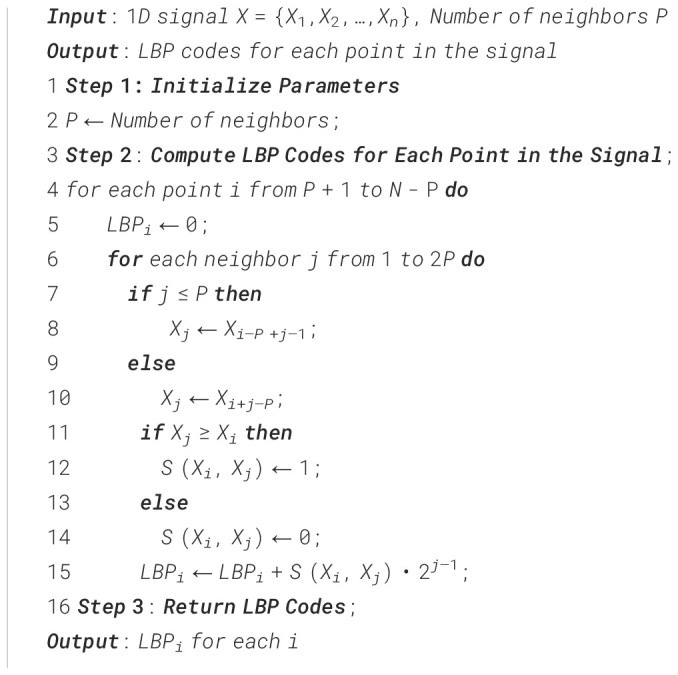

#### 2.4.3 Model Structure:

The **Table 1** below summarizes the modified structure of EfficientNet-LITE, detailing the input and output shapes at each stage, along with the expansion factors, repeat times, and strides.

**Table 1:** Structure of the proposed model.

Operators (modules)	Input shapes	Expansion factor	Output shapes	Repeat times	Strides
Input Layer	224 × 224 × 3	–	224 × 224 × 3	1	–
Conv2d	224 × 224 × 3	–	112 × 112 × 32	1	2
BatchNorm	112 × 112 × 32	–	112 × 112 × 32	1	–
Swish Activation	112 × 112 × 32	–	112 × 112 × 32	1	–
MBConv1	112 × 112 × 32	1	112 × 112 × 16	1	1
MBConv6	112 × 112 × 16	6	56 × 56 × 24	2	2
MBConv6	56 × 56 × 24	6	28 × 28 × 40	2	2
MBConv6	28 × 28 × 40	6	14 × 14 × 80	3	2
MBConv6	14 × 14 × 80	6	14 × 14 × 112	3	1
MBConv6	14 × 14 × 112	6	7 × 7 × 192	4	2
MBConv6	7 × 7 × 192	6	7 × 7 × 320	1	1
Conv2d 1 × 1	7 × 7 × 320	–	7 × 7 × 1280	1	1
Globalpool	7 × 7 × 1280	–	1 × 1280	1	–
Channel Attention	1 × 1280		1 × 1280	1	–
1-D LBP	1 × 1280		1 × 1290	1	
Dropout	1290	–	1290	1	–
Output Layer	1290	–	num_classes	1	–

The proposed EfficientNet-LITE model was meticulously engineered with a systematic arrangement of layers to attain a compromise between computing efficiency and performance. The input layer received potato leaf pictures measuring 224×224×3, which were subsequently processed through a Conv2D layer that downsampled the input to 112×112×32 with a stride of 2, thus diminishing the spatial dimensions while augmenting the channel depth. Batch Normalization and Swish Activation are utilized to stabilize and non-linearly activate the refined feature maps, priming them for the ensuing MBConv blocks.

The Swish activation function is defined Equation 5 as:


(5) $$Swish\left(x\right)=x\cdot \sigma \left(x\right)$$

where $\sigma \left(x\right)$ is the sigmoid function, given by Equation 6:


(6) $$\sigma \left(x\right)=\frac{1}{1+e^{-x}}$$

The MBConv layers facilitate effective feature extraction by gradually diminishing spatial dimensions while augmenting the amount of channels, culminating in a dense and compact feature representation. The model subsequently employed a 1x1 convolution to refine the features, followed by global pooling and a Channel Attention mechanism, which improved the model’s capacity to concentrate on the most pertinent channels. This was succeeded by a 1-D Local Binary Pattern (LBP) layer that expanded the feature vector to 1290 dimensions by integrating texture features.

#### 2.4.4 Performance Comparison: EfficientNet-LITE vs EfficientNet-B0

In deep learning, determines like Floating Point Operations (FLOPs), parameter count, model size, and depth are essential for evaluating the performance and efficiency of neural network models. FLOPs measure a model’s computational complexity, whereas the parameter count reflects its ability to learn and express intricate aspects. The model’s size pertains to storage demands, whereas depth frequently associates with the model’s capacity to discern complex patterns within the data.

EfficientNet-LITE had 359.69 MFLOPs, somewhat less than EfficientNet-B0’s 390. EfficientNet-LITE required fewer computational resources due to its lower FLOPs, making it ideal for mobile or edge devices. Despite adding Channel Attention and 1-D LBP features, EfficientNet-LITE maintained a computational efficiency similar to EfficientNet-B0, demonstrating its design efficiency. There are 3.11 million parameters in EfficientNet-LITE, compared to 5.3 million in B0. EfficientNet-LITE’s reduced parameters indicate a more streamlined architecture for memory-constrained applications. EfficientNet-LITE’s 12.46 MB model size was lower than EfficientNet-B0’s 20 MB due to fewer parameters. The compactness of EfficientNet-LITE accelerated model loading, memory usage, and inference times, making it better for real-time applications. **Table 2** shows the size of pre-trained network model.

**Table 2:** The model size of the main networks.

Networks	Model size	Parameters	Depth
VGG16	528 MB	138 million	23
Inception V3	92 MB	23.8 million	159
ResNet50	98 MB	25.6 million	–
DenseNet121	33 MB	8.1 million	121
MobileNet-V1	16 MB	4.2 million	88
MobileNet-V2	14 MB	3.5 million	88
NASNetMobile	23 MB	5.2 million	–
EfficientNet-B0	20 MB	5.3 million	24
**EfficientNet-LITE**	**12.46 MB**	**3.11 million**	**27**

Bold values indicate the best performance.

Also important is model depth, as deeper models can learn complex representations. EfficientNet-LITE had 27 layers, compared to 24 for EfficientNet-B0. This increased depth suggested that EfficientNet-LITE could capture more complex data characteristics, improving performance in sophisticated feature extraction tasks. The comparable FLOPs show that the extra depth did not reduce computing efficiency. EfficientNet-LITE balanced computational efficiency with model capacity. EfficientNet-LITE was ideal for mobile or embedded systems with limited computational resources because to its low FLOPs, parameter count, and model size. Despite being smaller, the model’s depth let it accomplish complex tasks well.

Finally, EfficientNet-LITE has fewer parameters (3.11 million) and a smaller model (12.46 MB vs. 20 MB) (Ubaidillah et al., 2022) than EfficientNet-B0. It has more layers (27 vs. 24) but fewer FLOPs (359.69 vs. 390), requiring fewer computations. EfficientNet-LITE was more resource-efficient and performed well.

### 2.5 KE-SVM optimization (kernel ensemble SVM optimization)

SVMs were widely employed in image classification and machine learning to define class boundaries. By translating input information into high-dimensional spaces, SVM classifiers (Sorensen and Nielsen, 2018) accurately handled complex and non-linear patterns in many applications.

However, datasets from uncontrolled environments with different backdrops, perspectives, and lighting conditions were difficult. Inconsistencies in image acquisition caused SVM kernels to struggle. Ensemble approaches (Sorensen and Nielsen, 2018) in machine learning improve performance by combining different models. This helped classify potato leaf diseases, where the dataset’s unpredictability required a more robust technique.

**Figure 5:**
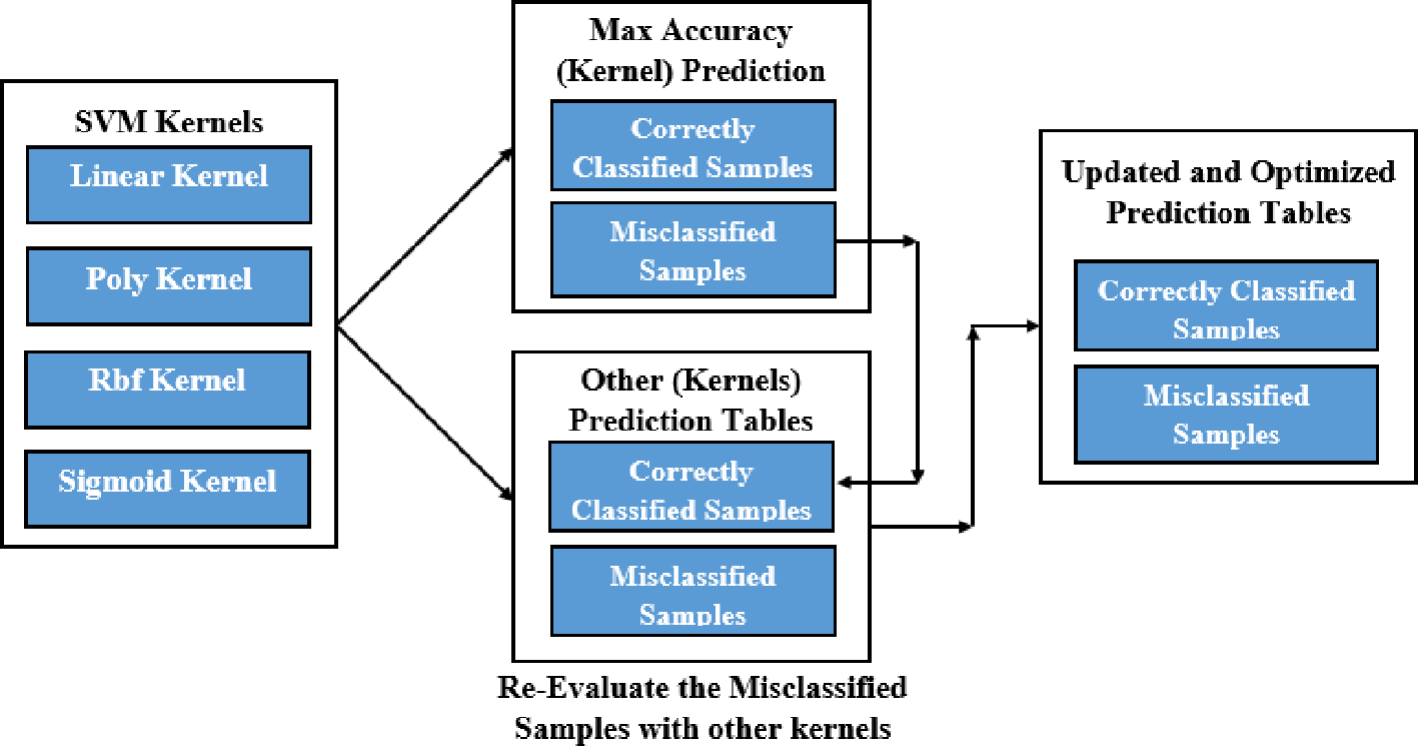
Block diagram of KE-SVM optimization.

**Figure 6:**
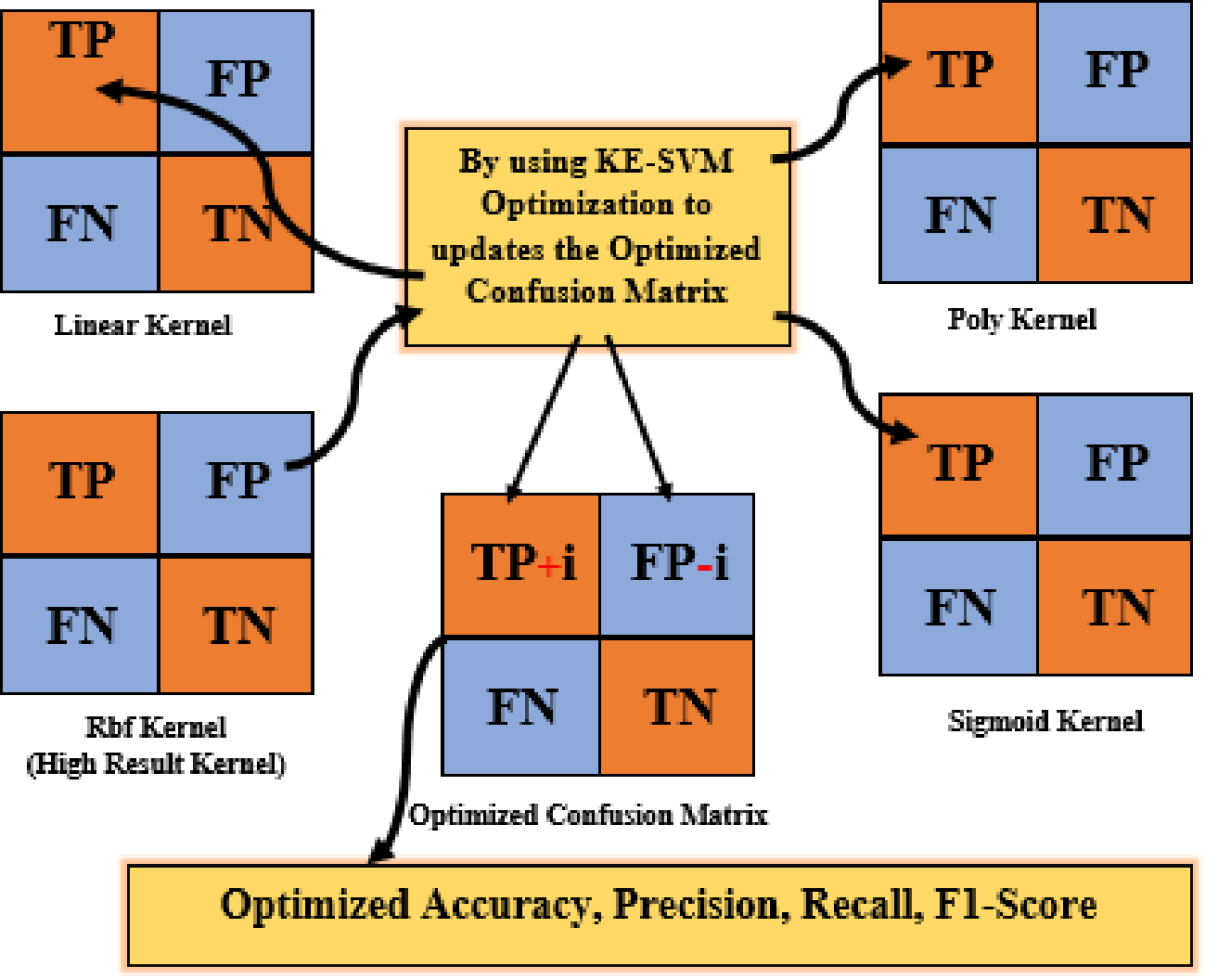
Misclassified samples re-evaluate with other kernels.

Kernel-Ensemble SVM (KE-SVM) Optimization used Linear, Polynomial, Radial Basis Function (RBF), and Sigmoid SVM kernels to address these issues. KE-SVM Optimization enhanced classification accuracy and discussed dataset variability by capturing different data features and integrating their predictions. KE-SVM Optimization improves classification by combining SVM kernel strengths. **Figures 5**, **6** shows the work flow of KE-SVM method. This method compares misclassified instances in one kernel against proper classifications in others. The optimum confusion matrix is iteratively adjusted using this ensemble technique to optimize classification accuracy across all classes.

The novelty of this work lies in the application of Kernel-Ensemble SVM (KE-SVM) Optimization (**Algorithm 2**) to substantially improve classification efficacy by harnessing the advantages of several SVM kernels. Misclassified samples from the kernel exhibiting the highest accuracy were verified against predictions from alternative kernels, with those accurately classified by other kernels deemed as True Positives. The iterative modification process persisted until all classes were sufficiently addressed, resulting in significant enhancements in classification performance.

Algorithm 2 **KE–SVM Optimization.**

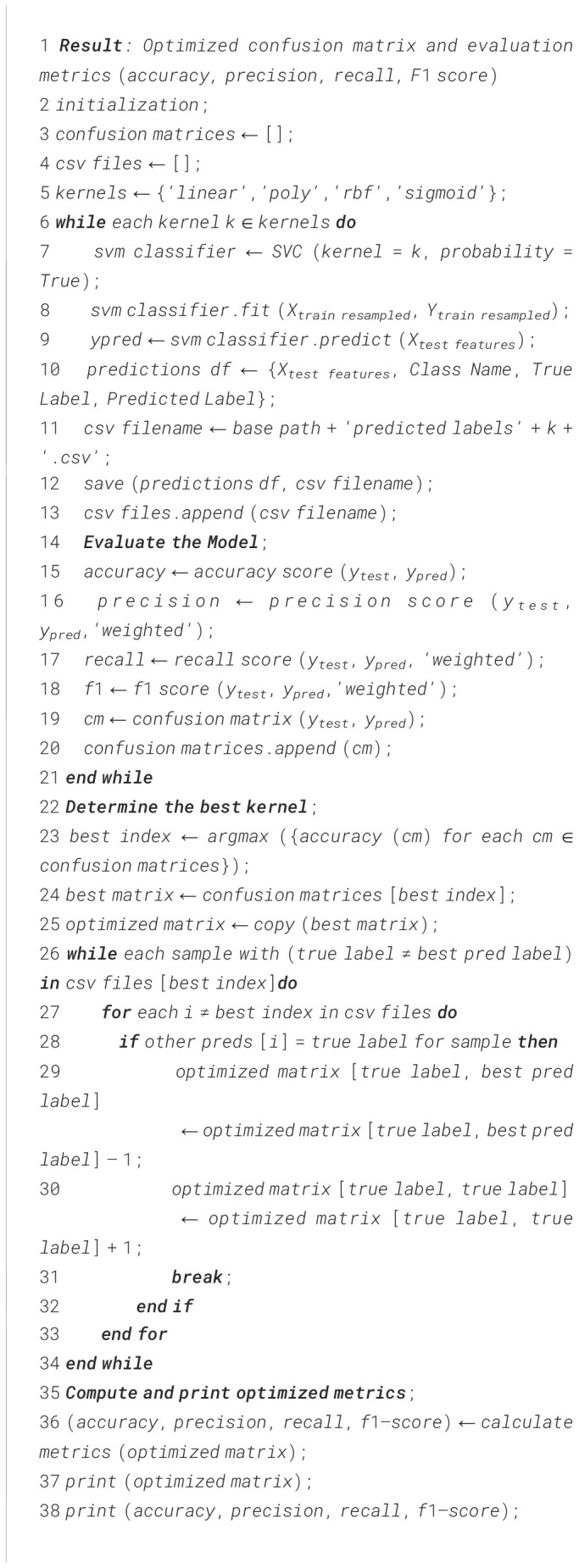

The potato leaf disease dataset, obtained from uncontrolled conditions, demonstrated that the SVM RBF kernel initially gave the highest performance among the kernels, attaining an accuracy of 79.38%. The Linear kernel achieved an accuracy of 72.89%, followed by the Polynomial kernel at 71.27%, and the Sigmoid kernel at 64.12%. The classification metrics and confusion matrix indicated a necessity for enhancement owing to the dataset’s heterogeneity, including differing backdrops and lighting conditions.

The SVM classifiers with different kernels attained good accuracy on the lab-controlled dataset from PlantVillage (potato species). The Polynomial kernel attained the maximum accuracy of 99.07%, succeeded by the RBF kernel at 98.84%, the Linear kernel at 98.38%, and the Sigmoid kernel at 96.06%. The classification report indicated an exceptional performance, with an overall accuracy of 1.00. The precision, recall, and F1-scores were remarkably elevated across all categories, indicating the consistent conditions of the dataset. The confusion matrix revealed minimal misclassifications, illustrating the effectiveness of the SVM Polynomial kernel in controlled laboratory circumstances.

The EfficientNet-LITE + SVM model demonstrated higher performance on datasets from both controlled and uncontrolled settings. Following KE-SVM optimization, the accuracy on the PlantVillage dataset rises to 99.54%, while on the uncontrolled environment dataset, it dramatically climbs to 87.82%, showing the model’s improved capacity to manage intricate, uncontrolled conditions.

## 3 Result and discussions

This study’s results are structured into three primary stages: (1) results before augmentation, (2) results before optimization, and (3) results after KE-SVM optimization. These stages comprehensively illustrate the progression in performance of the SVM classifiers when applied to controlled (PlantVillage) and uncontrolled environment datasets for diagnosing potato leaf diseases. The evaluation metrics employed include accuracy, precision, recall, F1-score, and other relevant measures to validate the model’s effectiveness. Equation 7, Equation 8, Equation 9, Equation 10 employed to calculate these measures were included to clarify the evaluation procedure.


(7) $$Accuracy=\frac{TP+TN}{TP+TN+FP+FN}$$

(8) $$Precision=\frac{TP}{TP+FP}$$

(9) $$Recall=\frac{TP}{TP+FN}$$

(10) $$F1\;Score=\frac{2*Precision*Recall}{Precision+Recall}$$

Accuracy evaluated overall correctness, whereas precision and recall examined the management of false positives and negatives. The F1-score offered a comprehensive assessment of the model’s classification performance, as illustrated in **Table 3** below.

**Table 3:** Shows the results of both datasets before optimization.

Model	Dataset	Accuracy	Precision	Recall	F1-score
EfficientNet-LITE + SVM	Potato Leaf Disease in Uncontrolled Environment	79.38%	80%	79%	79%
EfficientNet-LITE + SVM	PlantVillage (Potato Species)	99.07%	99%	99%	99%

The initial experiments were conducted using the raw dataset without applying Sobel edge filtering or augmentation techniques. The SVM classifier’s performance in uncontrolled and controlled environments revealed significant room for improvement. In the uncontrolled environment dataset, the accuracy was 75.62%, while in the lab-controlled dataset, the accuracy was 98.62%. These results underscore the challenges posed by the inherent variability in the uncontrolled environment dataset.

The lab-controlled dataset demonstrated high accuracy due to reduced variability and noise. Following data augmentation with Sobel edge filtering to enhance feature extraction, the performance of the SVM classifiers was evaluated before applying the KE-SVM optimization technique. The augmented samples contributed to improved classification, particularly in uncontrolled environments.

**Figure 7:**
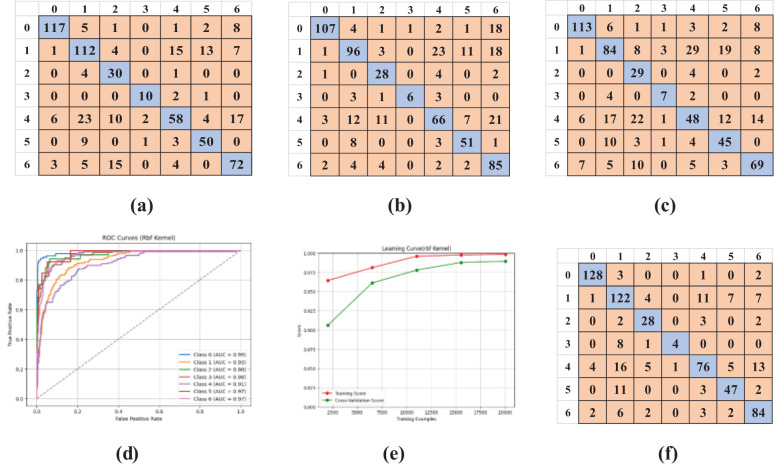
Confusion matrices of SVM kernels, AUC-ROC curve, and learning curve for the kernel with maximum accuracy (RBF). **(a)** Linear, **(b)** Polynomial, **(c)** Sigmoid, **(d)** AUC-ROC Curve, **(e)** Learning Curve, **(f)** RBF.

A comprehensive examination of the SVM model was performed on the uncontrolled environment dataset utilizing four distinct kernels: Linear (**Figure 7a**), Polynomial (**Figure 7b**), RBF (**Figure 7f**), and Sigmoid (**Figure 7c**). Confusion matrices were produced for each kernel, offering insights into the model’s classification proficiency across diverse categories: 0: Virus, 1: Phytophthora, 2: Nematode, 3: Fungi, 4: Bacteria, 5: Pest, 6: Healthy. Visual representations of these matrices are provided to illustrate the model’s performance.

The overall effectiveness was evaluated by plotting the AUC-ROC curve (**Figure 7d**) and learning curves (**Figure 7e**) for the kernel exhibiting the highest accuracy. These visuals facilitated awareness of the model’s capacity to generalize to unfamiliar data. To test the model’s dependability, 5-fold cross-validation was employed. **Table 4** results demonstrated constant performance across the folds, signifying the resilience of the SVM with RBF kernel, which attained the best accuracy.

**Table 4:** 5-Fold cross validation for potato leaf disease in uncontrolled environment dataset.

Fold	Training Accuracy	Validation Accuracy	Training Loss	Validation Loss
Fold 1	0.993	0.9656	0.0796	0.4502
Fold 2	0.9932	0.969	0.0726	0.3942
Fold 3	0.9933	0.9736	0.0705	0.302
Fold 4	0.9936	0.9702	0.0626	0.3604
Fold 5	0.993	0.9686	0.0757	0.3837
**Average**	**0.9932**	**0.9694**	**0.0722**	**0.3781**

Bold values indicate the best performance.

The SVM model utilizing the RBF kernel exhibited robust performance, attaining an average training accuracy of 99.32 and a validation accuracy of 96.94. The minor discrepancy between these metrics signified effective generalization throughout the sample. The uniformity of results over the five folds further emphasized the model’s resilience, even in an uncontrolled setting. The RBF kernel effectively captured intricate correlations within the data, demonstrating its appropriateness for the dataset’s inherent unpredictability. The model’s excellent accuracy underscored its efficacy in classifying leaf diseases.

**Figure 8:**
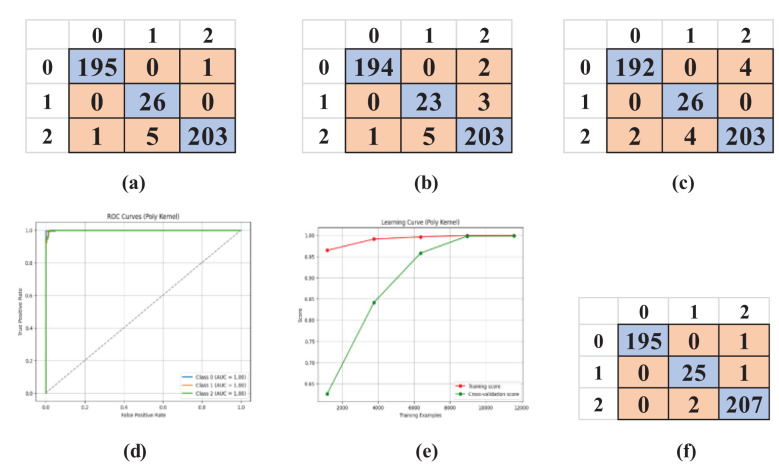
Confusion matrices of SVM kernels, AUC-ROC curve, and learning curve for the kernel with maximum accuracy (Polynomial). **(a)** Linear, **(b)** RBF, **(c)** Sigmoid, **(d)** AUC-ROC Curve, **(e)** Learning Curve, **(f)** Polynomial.

In the lab-controlled dataset, identical SVM kernels were utilized, and confusion matrices were produced for each kernel: Linear (**Figure 8a**), Polynomial (**Figure 8f**), RBF (**Figure 8b**), and Sigmoid (**Figure 8c**). It offers insights into the model’s categorization proficiency across different categories: 0: Early Blight, 1: Healthy, 2: Late Blight. The findings from this dataset exhibited remarkably high accuracy owing to the controlled environment, which minimized data fluctuation.

The model’s performance was additionally assessed by plotting the AUC-ROC curve (**Figure 8d**) and the learning curve (**Figure 8e**) for the optimal kernel. These curves demonstrated nearly flawless generalization. Consistent with the uncontrolled dataset, 5-fold cross-validation validated the model’s reliability, with **Table 5** indicating minimal variance among the folds.

**Table 5:** 5-Fold cross validation for PlantVillage dataset (potato species).

Fold	Training Accuracy	Validation Accuracy	Training Loss	Validation Loss
Fold 1	0.9996	1.0000	0.0004	0.000
Fold 2	0.9997	0.9972	0.0003	0.0028
Fold 3	0.9997	0.9986	0.0003	0.0024
Fold 4	0.9996	0.9979	0.0004	0.0031
Fold 5	0.9996	0.9983	0.0004	0.0038
**Average**	**0.9996**	**0.9984**	**0.0004**	**0.0024**

Bold values indicate the best performance.

The SVM model utilizing a polynomial kernel was assessed on laboratory-controlled data, demonstrating superior performance across all five folds. The model attained an average training accuracy of 99.96 and a validation accuracy of 99.84. The training loss of 0.0004 and validation loss of 0.0024 signify little error and robust generalization in a regulated environment. The results highlight the efficacy of the polynomial kernel in managing clean, organized data, exhibiting little variability relative to uncontrolled contexts.

### 3.1 After optimization

**Figure 9:**
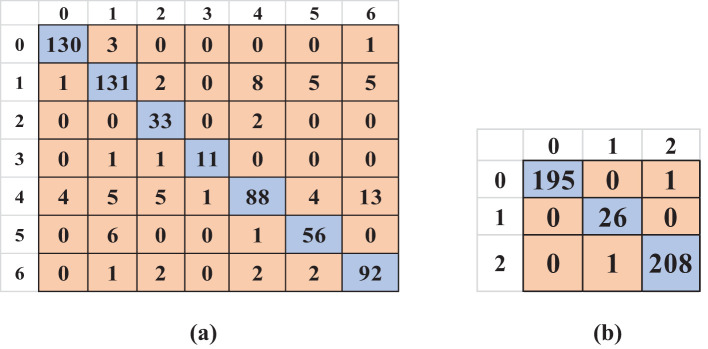
Optimized confusion matrices for both datasets: **(a)** Uncontrolled data and **(b)** Laboratory-controlled data.

After implementing KE-SVM Optimization, the model’s performance on the uncontrolled environment dataset shown significant enhancement. The optimal accuracy increased to 87.82%, accompanied by enhancements in precision to 86.77%, recall to 88.18%, and F1-score to 87.19%. The lab-controlled dataset has been somewhat enhanced to 99.54%. (**Figure 9a**) presents the optimized confusion matrix for uncontrolled data, while (**Figure 9b**) displays the matrix for the laboratory-controlled dataset.

The optimization approach improved classification by cross-validating misclassified examples from the most effective kernel with predictions from other kernels, resulting in a more precise and balanced confusion matrix. **Table 6** presents the optimal outcomes of the KE-SVM optimization technique.

**Table 6:** Optimized results for both lab and uncontrolled dataset.

Model	Dataset	No. of Classes	Accuracy
EfficientNet-LITE + SVM	Potato Leaf Disease in Uncontrolled Environment	07	87.82%
EfficientNet-LITE + SVM	PlantVillage(Potato Species)	03	99.54%

### 3.2 Comparative performance

The proposed model (EfficientNet-LITE + KE-SVM Optimization) exhibited substantial enhancements in accuracy relative to previous models utilized on comparable datasets. Prior to optimization, the model attained an accuracy of 79.38%, which rose to 87.82% post-optimization. This performance surpassed those of models like DenseNet121, ResNet50, and MobileNetV3-Large, which exhibited accuracies between 59.16% and 73.63%. This significant enhancement can be ascribed to the ensemble SVM kernel methodology and improved feature extraction with EfficientNet-LITE.

In the lab-controlled PlantVillage dataset, the suggested model attained nearly flawless accuracy both prior to and subsequent to KE-SVM Optimization. The model initially achieved an accuracy of 99.07%, which then increased to 99.54% during optimization. This performance surpassed other prominent models, including ResNet152, InceptionV3, and VGNet, which exhibited accuracies between 95.24% and 98.34%. The substantial enhancement upon optimization is attributable to the improved feature extraction and the strong classification capabilities of KE-SVM.

An optimized version of EfficientNetB0, EfficientNet-LITE, integrated Channel Attention (CA) and 1D Local Binary Pattern (LBP) features to increase feature extraction. This model prioritized potato leaf traits while being computationally efficient, making it ideal for resource-constrained mobile devices. KE-SVM Optimization used linear, polynomial, RBF, and sigmoid kernels to overcome typical SVM constraints. With SMOTE and confusion matrix optimization, classification accuracy improved, handling class imbalance and data variability.

The strengths of EfficientNet-LITE and KE-SVM Optimization were combined. EfficientNet-LITE’s superior feature extraction and KE-SVM Optimization’s classification created a model that could handle complex datasets. This collaboration produced high accuracy and reliable performance in all settings. The combined model exceeded expectations in early illness identification and uncontrolled environment management to satisfy research objectives. The results confirmed the model’s efficacy and versatility in solving research problems.

**Tables 7** and **8** highlight the superior performance of our proposed EfficientNet-LITE + KE-SVM Optimization model compared to existing methods. Notably, the model achieved an accuracy of 87.82% on uncontrolled datasets and 99.54% on the PlantVillage dataset, surpassing models such as DenseNet121 and ResNet50. These results underscore the robustness of our approach in handling variability and improving classification accuracy. The enhanced classification accuracy of our model has significant implications for agricultural diagnostics. By addressing challenges posed by uncontrolled environments, our model paves the way for reliable and resource-efficient solutions applicable in real-world farming scenarios. This contributes to the broader goal of precision agriculture and early disease detection.

**Table 7:** To compare the results with existing state-of-art-methods for uncontrolled dataset.

Author & Year	Model Name	Dataset	Accuracy
Penghui Gui, et al., 2021	CNN	Field-PV	72.03%
A Ubaidillah, et al., 2022	ANN	Cotton Disease (Field Data)	74.44%
AANIS AHMAD, et al., 2023	DenseNet169(RGBA)	Field-PV	77.50%
Shabrina, et al., 2024	EfficientNetV2B3	Potato Leaf Disease in Uncontrolled Environment	73.63%
	MobileNetV3-Large		72.03%
	VGG-16		59.81%
	ResNet50		68.17%
	DenseNet121		59.16%
**Proposed Model**	**EfficientNet-LITE(Before Optimization)**	Potato Leaf Disease in Uncontrolled Environment	**79.38%**
**Proposed Model**	**EfficientNet-LITE(After Optimization)**	Potato Leaf Disease in Uncontrolled Environment	**87.82%**

Bold values indicate the best performance.

**Table 8:** To compare the results with existing state-of-art-methods with PlantVillage(Potato) dataset.

Author & Year	Model Name	Dataset	Accuracy
Saeed Z et al., 2021	ResNet152	PlantVillage(Potato)	98.34%
	InceptionV3		95.24%
Rabia M et al., 2022	ResNet-202	PlantVillage(Potato)	97.2%
Shabrina et al., 2024	EfficientNetV2B3	PlantVillage(Potato)	98.15%
Jain J et al., 2024	EfficientNetB0	PlantVillage(Potato)	99.05
**Proposed Model**	**EfficientNet-LITE(Before Optimization)**	**PlantVillage(Potato)**	**99.07%**
**Proposed Model**	**EfficientNet-LITE(After Optimization)**	**PlantVillage(Potato)**	**99.54%**

Bold values indicate the best performance.

## 4 Conclusion

In conclusion, our research revealed the effectiveness of combining EfficientNet-LITE with KE-SVM Optimization for the classification of potato leaf diseases. Initially, SVM classifiers demonstrated disparate performance, with the RBF kernel achieving 79.38% accuracy on uncontrolled data and the sigmoid kernel reaching 99.07% accuracy on laboratory-controlled data. Subsequent to KE-SVM Optimization, the accuracy on the uncontrolled dataset markedly increased to 0.8782, with precision at 86.77%, recall at 88.18%, and F1-score at 87.19%. Conversely, the accuracy on the lab-controlled dataset exhibited a minor enhancement to 99.54%. This integrated model adeptly tackles issues associated with early disease classification, dataset variability, and model robustness, demonstrating its versatility and dependability across many settings. Future work could explore integrating more comprehensive datasets that combine image data with clinical parameters such as plant height, size, irrigation schedules, and expert farmer insights. Additionally, leveraging generative AI techniques could provide holistic solutions for farmers, enhancing decision-making and improving crop management practices.

## Data Availability

The datasets presented in this study can be found in online repositories. The names of the repository/repositories and accession number(s) can be found in the article/supplementary material.
